# Piezo type mechanosensitive ion channel component 1 facilitates gastric cancer omentum metastasis

**DOI:** 10.1111/jcmm.16217

**Published:** 2021-01-13

**Authors:** Xiaofei Wang, Guang Cheng, Yu Miao, Fangyuan Qiu, Lugen Bai, Zhongfei Gao, Yunning Huang, Liru Dong, Xing Niu, Xin Wang, Yuyang Li, Hui Tang, Yuanyi Xu, Xudong Song

**Affiliations:** ^1^ Department of Pathology North China University of Science and Technology Affiliated Hospital Tangshan China; ^2^ Central Laboratory of Clinical Medical College North China University of Science and Technology Affiliated Hospital Tangshan China; ^3^ Department of GI Medicine General Hospital of Ningxia Medical University Yinchuan China; ^4^ Department of Medical Jining Second People’s Hospital Jining China; ^5^ Department of laboratory Jingbian County People’s Hospital Yulin China; ^6^ Department of Medical Jining First People’s Hospital Jining China; ^7^ Department of Gastrointestinal Surgery People’s Hospital of Ningxia Hui Autonomous Region Yinchuan Ningxia China; ^8^ Department of Second Clinical College Shengjing Hospital Affiliated to China Medical University Shenyang, Liaoning China; ^9^ Department of Pathology Ningxia Medical University Yinchuan China

**Keywords:** Ca^2+^ influx, gastric cancer, HIF‐1α, migration, omentum metastasis, Piezo1

## Abstract

The peritoneum, especially the omentum, is a common site for gastric cancer (GC) metastasis. Our aim was to expound the role and mechanisms of Piezo1 on GC omentum metastasis. A series of functional assays were performed to examine cell proliferation, clone formation, apoptosis, Ca^2+^ influx, mitochondrial membrane potential (MMP) and migration after overexpression or knockdown of Piezo1. A GC peritoneal implantation and metastasis model was conducted. After infection by si‐Piezo1, the number and growth of tumours were observed in abdominal cavity. Fibre and angiogenesis were tested in metastatic tumour tissues. Piezo1 had higher expression in GC tissues with omentum metastasis and metastatic lymph node tissues than in GC tissues among 110 patients. High Piezo1 expression was associated with lymph metastasis, TNM and distant metastasis. Overexpressed Piezo1 facilitated cell proliferation and suppressed cell apoptosis in GC cells. Moreover, Ca^2+^ influx was elevated after up‐regulation of Piezo1. Piezo1 promoted cell migration and Calpain1/2 expression via up‐regulation of HIF‐1α in GC cells. In vivo, Piezo1 knockdown significantly inhibited peritoneal metastasis of GC cells and blocked EMT process and angiogenesis. Our findings suggested that Piezo1 is a key component during GC omentum metastasis, which could be related to up‐regulation of HIF‐1α.

## 1 INTRODUCTION

Gastric cancer (GC) is one of the most common gastrointestinal malignancies globally.^1^, ^2^ Tumour metastasis is a pivotal biological feature of GC and is also the main cause of deaths for GC patients. About 53%‐66% of distant metastatic GC occurs in the peritoneum, especially the omentum.^3^ When the GC cells spread to the abdominal cavity, they may grow in the abdominal viscera and omentum.^3^ Surgical treatment often fails to eradicate these cells. Therefore, patients’ prognosis is still poor. Postoperative chemotherapy can destroy metastasis and residual tumours, which has been considered as an effective treatment for postoperative GC metastasis.^4^ However, because of the presence of the peritoneal serosal barrier, most intravenous chemotherapy drugs cannot reach the abdominal cavity. Also, chemotherapy drugs have a small molecular weight and are easily absorbed in the abdominal cavity. Thus, the efficacy of postoperative chemotherapy is relatively low. However, the mechanism by which GC cells transform from abscission to omental metastasis is unclear.

Piezo type mechanosensitive ion channel component 1 (Piezo1) protein is a mechanically sensitive cation channel.^5^, ^6^ As morphological changes occur in epithelial cells, cell homeostasis becomes imbalanced, and Piezo1 is activated, thereby accelerating Ca^2+^ influx and cell cycle arrest.^7^, ^8^ In the early stages of tissue development, Piezo1 is activated by shearing force and Calpain is then activated in endothelial cells (ECs), leading to the division of focal adhesions and angiogenesis.^9^ Piezo1 can promote vascular EC migration by activation of Ca^2+^ channels, thereby driving embryonic angiogenesis.^10^ Cell mobility is a key characteristic to ensure that tumour cells invade surrounding tissues and occur distant metastasis. Hypoxia may enhance the migrant ability of tumour cells that is closely related to tumour metastasis. In the hypoxic tumour microenvironment, hypoxia inducible factor 1α (HIF‐1α), as a transcription factor, is notably elevated in tumour cells.^11^ More importantly, its up‐regulation can accelerate epithelial‐mesenchymal transition (EMT) and angiogenesis via vascular endothelial growth factor (VEGF), thereby promoting tumour growth and metastasis. Thus, HIF‐1α has been considered as an upstream regulatory factor of VEGF. Moreover, mitochondrial calcium ion influx could promote VEGF expression via up‐regulation of HIF‐1α, inducing angiogenesis.^12^ It has been found that Piezo1 could mediate HIF‐1α through Ca^2+^ influx, which contributes to cyclical hydrostatic pressure.^13^

In this study, we described the role and underlying molecular mechanisms of Piezo1 on GC omental implantation and metastasis. High Piezo1 expression was identified in GC tissues with omentum metastasis and metastatic lymph node tissues. Consistent with previous studies, Piezo1 could accelerate cell proliferation and migration in GC cells.^14^, ^15^ Furthermore, Piezo1 could promote cell migration and Calpain1/2 expression via up‐regulation of HIF‐1α in GC cells. In peritoneal metastatic GC mouse model, Piezo1 knockdown could notably inhibit peritoneal metastatic tumour growth, block EMT process and angiogenesis. Therefore, Piezo1 could become a potential therapeutic target for GC patients with omentum metastasis.

## 2 MATERIALS AND METHODS

### 2.1 Tissue specimens

In total, 110 formalin‐fixed and paraffin‐embedded gastric cancer specimens were gathered from North China University of Science and Technology Affiliated Hospital (Tangshan, Hebei, China) between March 2014 and March 2016. All cases did not receive radiochemotherapy or immunotherapy before surgery. Our study was in strictly line with the guidelines in the Declaration of Helsinki. All patients signed written informed consent. The project acquired the approval by the Ethics Committee of North China University of Science and Technology Affiliated Hospital (2014025).

### 2.2 Cell culture

Two human GC cell lines SNU‐1 and HGC‐27 that passed STR test were purchased from Beijing Beina Chuanglian Biotechnology Research Institute (Beijing, China), which were maintained in RPMI‐1640 medium plus 10% FBS (Gibco, New York, USA) in a 5% CO_2_ incubator at 37 ℃.

### 2.3 qRT‐PCR

Extracted RNA from tissues or cells was reverse transcribed into cDNA by a reverse transcriptase kit (Invitrogen, #K1622, USA). qRT‐PCR was presented via TB Green® Premix Ex Taq™ II kit (TAKARA, RR820A, Japan). The relative expression levels were quantified with the 2^−ΔΔCt^ method. Primer sequence information is listed in Table 1.

**TABLE 1:** Primers of target genes for qRT‐PCR

Target genes	Primer sequence (5′‐3′)
Piezo 1	F: 5′‐GGACTCTCGCTGGTCTACCT‐3′
	R: 5′‐GGGCACAATATGCAGGCAGA‐3′
HIF‐1α	F: 5′‐GAAAGCGCAAGTCTTCAAAG‐3′
	R: 5′‐TGGGTAGGAGATGGAGATGC‐3′
VEGF	F: 5′‐CTCGCAGTCGCGGAGA‐3′
	R: 5′‐GCAGCCTGGACCCTTGGC‐3′
Vimentin	F: 5′‐CGCTTCGCCAACACAT‐3′
	R: 5′‐AGGGCATCCACTTCACAG‐3′
N‐cadherin	F: 5′′‐CAACTTGCCAGAAAACTCCAGG‐3′
	R: 5′‐ATGAAACCGGGCTATCTGCTC‐3′
E‐cadherin	F: 5′‐GACGCGGACGATGATGTGAAC‐3′
	R: 5′‐TTGTACGTGGTGGGATTGAAG‐3′
GADPH	F: 5′‐CAAGGTCATCCATGACAACTTTG‐3′
	R: 5′‐GTCCACCACCCTGTTGCTGTAG‐3′

### 2.4 Western blot and co‐immunoprecipitation

Cells or tissues were extracted using RIPA lysis plus protease inhibitor on the ice. For co‐immunoprecipitation, cell lysates were extracted and incubated with 2 μg specific antibodies for 18 hours at 4°C. Then, samples were incubated with 50 μL protein G agarose beads for 3 hours. After washing the beads for 5 times by lysis buffer, the precipitated proteins were resuspended in 30 μL SDS sample buffer and boiled at 95°C for 10 minutes. Proteins were separated by SDS‐PAGE and transferred onto PVDF membrane (Merck Millipore, Germany). The membrane was blocked by 0.5% skimmed milk for 2 hours at room temperature. Then, the membrane was incubated with primary antibodies at 4 ℃ overnight, followed by incubation with secondary antibodies at room temperature for 2 hours. Primary antibodies were as follows: anti‐Piezo1 (1:500; ProteinTech, #15939‐1‐AP, China), anti‐Calpain1 (1:1000; Abcam, #ab108400, USA), anti‐Calpain2 (1:1000; Abcam, #ab39165), anti‐HIF‐1α (1:1000; Abcam, #ab16066), anti‐VEGF (1:1000; ProteinTech, #26381‐1‐AP), anti‐E‐cadherin (1:1000; ProteinTech, #20874‐1‐AP), anti‐P53 (1:500; ProteinTech #10442‐1‐AP), anti‐P21 (1:500; ProteinTech, #10355‐1‐AP), anti‐N‐cadherin (1:1000; Abcam, #ab76057), anti‐Vimentin (1:1000; Abcam, #ab92547), CDK4 (1/2000; Abcam, #ab226474), CDK6 (1/1000; Abcam, #ab151247), CyclinD1 (1/200; Abcam, #ab16663) and anti‐β‐actin (1:1000; Abcam, #ab179467). Protein blots were visualized using ECL luminescence kit.

### 2.5 Immunohistochemistry and immunofluorescence staining

For immunohistochemistry staining, formalin‐fixed and paraffin‐embedded specimens were incubated with primary antibodies at 4 ℃ overnight, including anti‐Piezo1 (1:100), anti‐Calpain1 (1:150), anti‐Calpain2 (1:150), anti‐HIF‐1α (1:100), Ki‐67 (1:100; ProteinTech, #27309‐1‐AP), anti‐VEGF (1:100), anti‐E‐cadherin (1:200) and anti‐Vimentin (1:200), followed by incubation with secondary antibodies (ZSGB‐BIO, #SPN‐9001, China) at room temperature for 1 hours. After adding DAB solution (Cell Signaling Technology, #8961s, USA), the sections were stained with haematoxylin for 2 minutes. The immunohistochemical score was assessed by two experienced pathologists under double blindness. The staining intensity was divided into no staining (0 points), light staining (1 point), moderate staining (2 points) and strong staining (3 points). The staining range was divided into the number of positive cells ≤10% (0 points), 10%< the number of positive cells ≤25% (1 point), 25%< the number of positive cells ≤50% (2 points) and the number of positive cells >50% (3 points). The immunohistochemical score was determined based on the staining intensity score multiplied by the staining range score.

For immunofluorescence staining, the sections were incubated with Piezo1 (1:150) and HIF‐1α (1:150) antibodies, followed by incubation with Alexa Fluor® 488 Conjugate (ZSGB‐BIO, #ZF‐0512, 1:100, China) and Alexa Fluor® 594 Conjugate (ZSGB‐BIO, #ZF‐0513, 1:100). The results were examined under a fluorescence microscope (BX61, Olympus Corporation, Tokyo, Japan) at magnifications of ×400 and ×1000.

### 2.6 Cell counting kit‐8 (CCK‐8) assay

Cells were treated with different concentrations of Yoda1 (12.5, 50, 100, 200 and 400 µmol/L; Cayman Chemical, #448947‐81‐7, USA) for 48 hours. Cell viability was assessed using CCK‐8 kit. Briefly, the cells were seeded into 96‐well plates (1000 cells/well) for 24 hours and were incubated with 10 μL CCK‐8 reagent for 2 hours in strictly line with the manufacturer's instructions. OD value was determined at a wavelength of 450 nm.

### 2.7 Transfection

The small interfering RNA (siRNA) oligonucleotides for Piezo1 were purchased from Sangon Biotech (R849, Shanghai, China). The two siRNAs targeting human Piezo1 were as follows:siRNA‐1, 5′‐CCAAGAAGUACAAUCAUCUCA‐3′ (sense), 5′‐AGAUGAUUGUACUUCUUGGTG‐3′ (antisense); siRNA‐2, 5′‐GACUACUUCCUGUUUGAGUCC‐3′ (sense), 5′‐ACUCAAACAGGAAGUAGUCCC‐3′ (antisense). GFP‐PURO lentiviral short hairpin RNAs (shRNAs) targeting human HIF1α (GFP‐PURO‐HIF1α) were purchased from Open Biosystems (HANBIO, Shanghai, China). Transfection was performed using Lipofectamine 2000 Transfection Reagent (Invitrogen, #11668019, USA).

### 2.8 Clone formation assay

Cells were inoculated into 6‐well plates (1000 cells/well), followed by 10 days’ culture. Cells were then fixed with paraformaldehyde for 15 minutes and stained with 1% crystal violet for 20 minutes. The number of cells was counted under a microscope (Olympus, Japan).

### 2.9 Flow cytometry for apoptosis assay

After treatment with different concentrations of Yoda1 (12.5, 50, 100, 200 and 400 µmol/L) for 48 hours, apoptotic cells were examined using Annexin V‐FITC/7PI apoptosis kit in line with the manufacturer's instructions.

### 2.10 Flou‐4 AM Ca^2+^ imaging and mitochondrial membrane potential (MMP)

Ca^2+^ levels were measured using Fluo4‐AM (Invitrogen, #F14201, USA). MMP was detected using MMP detection kit (JC‐1; Beyotime, China) in accordance to the manufacturer's instructions. The prepared JC‐1 staining working solution was added to a 6‐well plate (500 μL/well). The supernatant was discarded after centrifugation at 1500 *g* for 5 minutes at 4°C. JC‐1 staining was observed under a fluorescence microscope.

### 2.11 Transwell assay

30 μL culture medium containing Matrigel was added into the upper layer of transwell chamber (Millipore, Massachusetts, USA). After gel, 50 μL culture medium plus 5% BSA was added to each well for 30 minutes at 37 ℃. Then, the medium was removed. 3 × 10^3^ starved of cells for 12 hours were seeded onto each well pre‐filled with 500 μL complete culture medium plus 10% FBS and maintained for 36 hours. Then, cells were fixed with 4% paraformaldehyde for 20 minutes and stained with 0.1% crystal violet for 5 minutes. The number of cells was counted under a microscope.

### 2.12 Wound healing assay

Cells were seeded onto 6‐well plates. When the cell confluence reached 80%, the cells were scratched with a 200 μm pipette tip. Then, the cells were incubated in a serum‐free medium for 48 hours and the results were observed under a microscope.

### 2.13 Experimental animals

A total of 10 male BALB/c nude mice (5‐6 weeks old, 18‐22 g) were purchased from Beijing Vital River Laboratory Animal Technology Co., Ltd. (Beijing, China). All procedures were performed in accordance with the animal protocol approved by the North China University of Science and Technology Laboratory Animal Centre and internationally recognized guidelines on animal welfare.

### 2.14 Construction of a nude mouse model of GC peritoneal metastasis

All nude mice were randomly assigned to two groups, including control group (n = 5) and si‐Piezo1 group (n = 5). The nude mice were anaesthetized by 0.5% pentobarbital sodium, which were intraperitoneally injected with 300 μL serum‐free medium resuspended cell suspension (containing 5 × 10^6^ HGC‐27 cells). Two week later, 5 nude mice in the si‐Piezo1 group were injected intraperitoneally with 25 mg/kg si‐Piezo1 twice a week. Corn oil was used to dissolve si‐Piezo1. Five nude mice in the control group were injected intraperitoneally with corn oil twice a week. The growth status and bodyweight of all nude mice were recorded daily. After four weeks, the nude mice were killed by excess pentobarbital sodium. The tumours in the abdominal cavity of nude mice were removed and used for pathological examination or other assays. This experiment was approved by the Animal Care Committee of our institute (2014025).

### 2.15 Masson's Trichrome and reticular fibre staining

Masson's trichrome staining of sections was presented by Masson's Trichrome Staining Kit (Solarbio, #M1340, China). For reticular fibre staining, potassium permanganate was used to incubate the sections for 3 minutes, followed by oxalic acid and ammonium ferric sulphate for 1 minutes. Then, the sections were treated with Silver ammonia for 5 minutes and formaldehyde solution for 30 second. After that, the sections were incubated with gold chloride solution for 5 minutes and sodium thiosulphate for 2 minutes. The sections were stained by Eosin staining solution for 30 seconds. Images were analysed using Image‐Pro Plus 6.0 software (Media Cybernetics, Inc, Rockville, MD, USA).

### 2.16 Statistical analysis

The data were analysed using SPSS 23.0 software (IBM SPSS, Armonk, NY, USA) and GraphPad Prism 8.0 (GraphPad, San Diego, CA). Each assay was repeated in triplicate. Data were expressed as mean ± standard deviation (SD). The comparison of two groups was carried out by student's *t* test, whereas multiple comparisons were analysed using ANOVA. The correlation between Piezo1 expression and clinical parameters of GC was analysed using Chi test. The Pearson's correlation analysis of the two factors was presented. *P* < .05 indicates statistically significant.

## 3 RESULTS

### 3.1 Piezo1 is highly expressed in GC tissues with omentum metastasis

**FIGURE 1:**
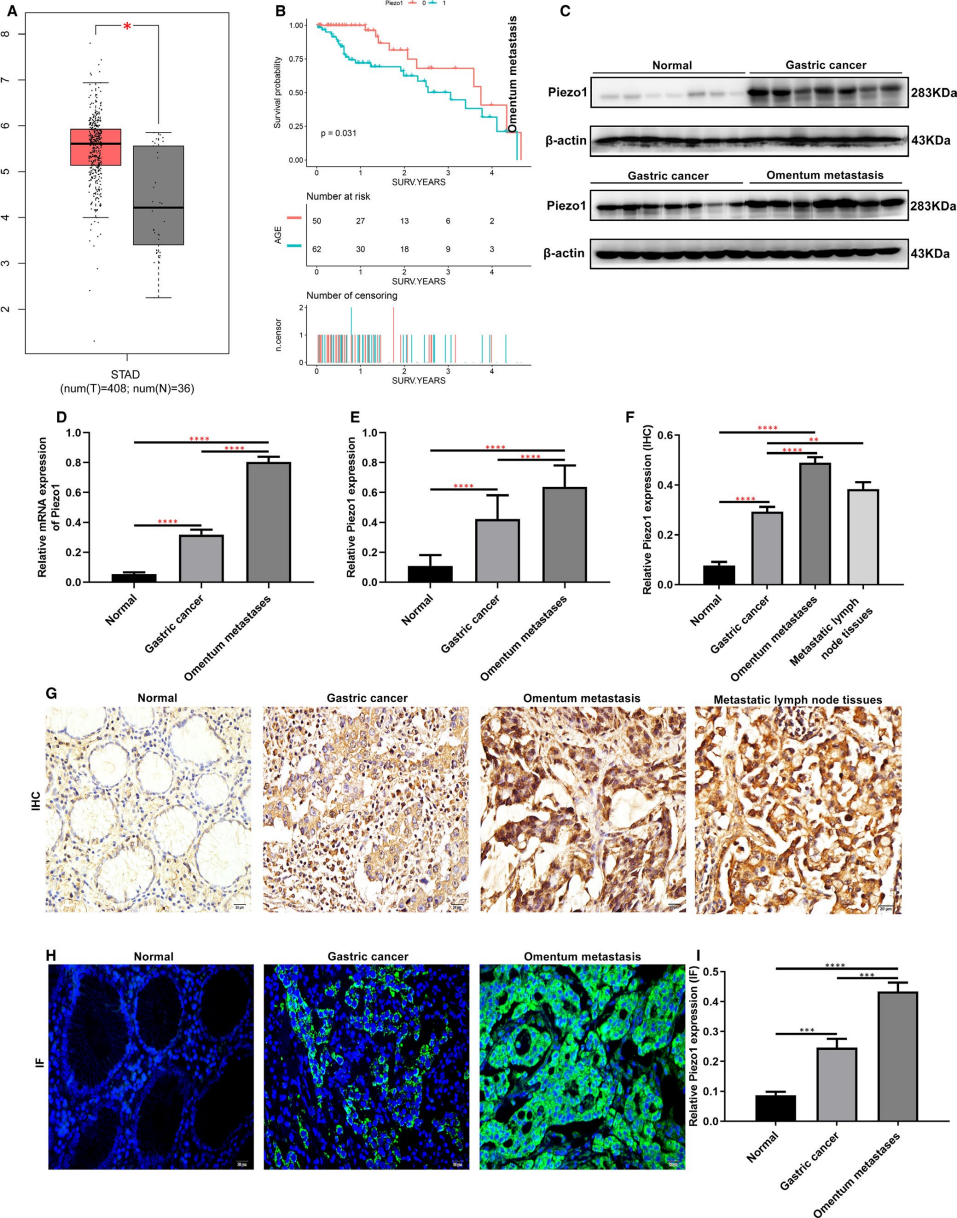
High Piezo1 expression in GC tissues along with omentum metastasis. A, Piezo1 was highly expressed in GC tissues than in normal tissues. Red represents gastric cancer samples, and grey represents normal samples. B, Overall survival analysis suggested that high Piezo1 expression predicted poorer overall survival time for patients with GC. C‐E, Western blot and qRT‐PCR assays were used to examine the expression of Piezo1 in normal tissues, GC tissues and GC tissues with omentum metastasis. F‐I, Immunohistochemistry and immunofluorescence results showed the expression and distribution of Piezo1 normal tissues, GC tissues, GC tissues with omentum metastasis or metastatic lymph node tissues. *P < .05; **P < .01; ***P < .001; ****P < .0001

In accordance with the gene expression profiling interactive analysis (GEPIA) database (http://gepia.cancer‐pku.cn), the expression of Piezo1 in GC tissues was distinctly higher than that in normal tissues (Figure 1A). Totally, 110 GC patients were recruited in our study, divided into high‐ and low‐expression groups based on the median value of Piezo1 expression. Overall survival (OS) analysis results suggested that Piezo1 was negatively correlated with shorten OS time of gastric cancer patients (Figure 1B). Also, Piezo1 expression was in association with lymphatic metastasis and with TNM staging (Table 2). At the mRNA and protein levels, Piezo1 expression was significantly higher in GC tissues than that in normal tissues (Figure 1C‐E). A higher expression level of Piezo1 was found in GC tissues with omentum metastasis in comparison with GC tissues (Figure 1C‐E). Furthermore, immunohistochemistry and immunofluorescence staining were separately performed to detect the expression and location of Piezo1 in GC tissues. The results demonstrated that that Piezo1 was primarily expressed in the cell membrane and cytoplasm of GC cells (Figure 1F‐I). As expected, the quantitative data showed that there was a higher expression level of Piezo1 in GC tissues than normal tissues. Also, in comparison with GC tissues, Piezo1 expression was notably elevated in GC tissues with omentum metastasis and metastatic lymph node tissues (Figure 1F‐G).

**TABLE 2:** The correlation between Piezo1 expression and clinicopathological features of GC

Clinical parameters	Total (n = 110)	Piezo1		*χ* ^2^	*P*
		Positive	Negative		
Gender					
Male	65	47	8	12.024	0.002^**^
Female	45	32	23		
Age					
<60	66	48	11	6.705	0.035^*^
≥60	44	31	20		
Depth of invasion					
T1/T2	42	26	13	0.955	0.620
T3/T4	68	53	18		
Lymph metastasis					
N0	38	24	21	14.176	0.001^***^
N1/N2/N3	72	55	10		
TNM stage					
Ⅰ‐Ⅱ	39	26	23	17.703	0.001^***^
Ⅲ‐Ⅳ	71	53	8		
Distant metastasis					
Absent	43	28	19	6.454	0.040^*^
Present	67	51	12		

* *P* < .05;

** *P* < .01;

*** *P* < .001.

### 3.2 Piezo1 facilitates cell proliferation and suppresses cell apoptosis in GC cells

**FIGURE 2:**
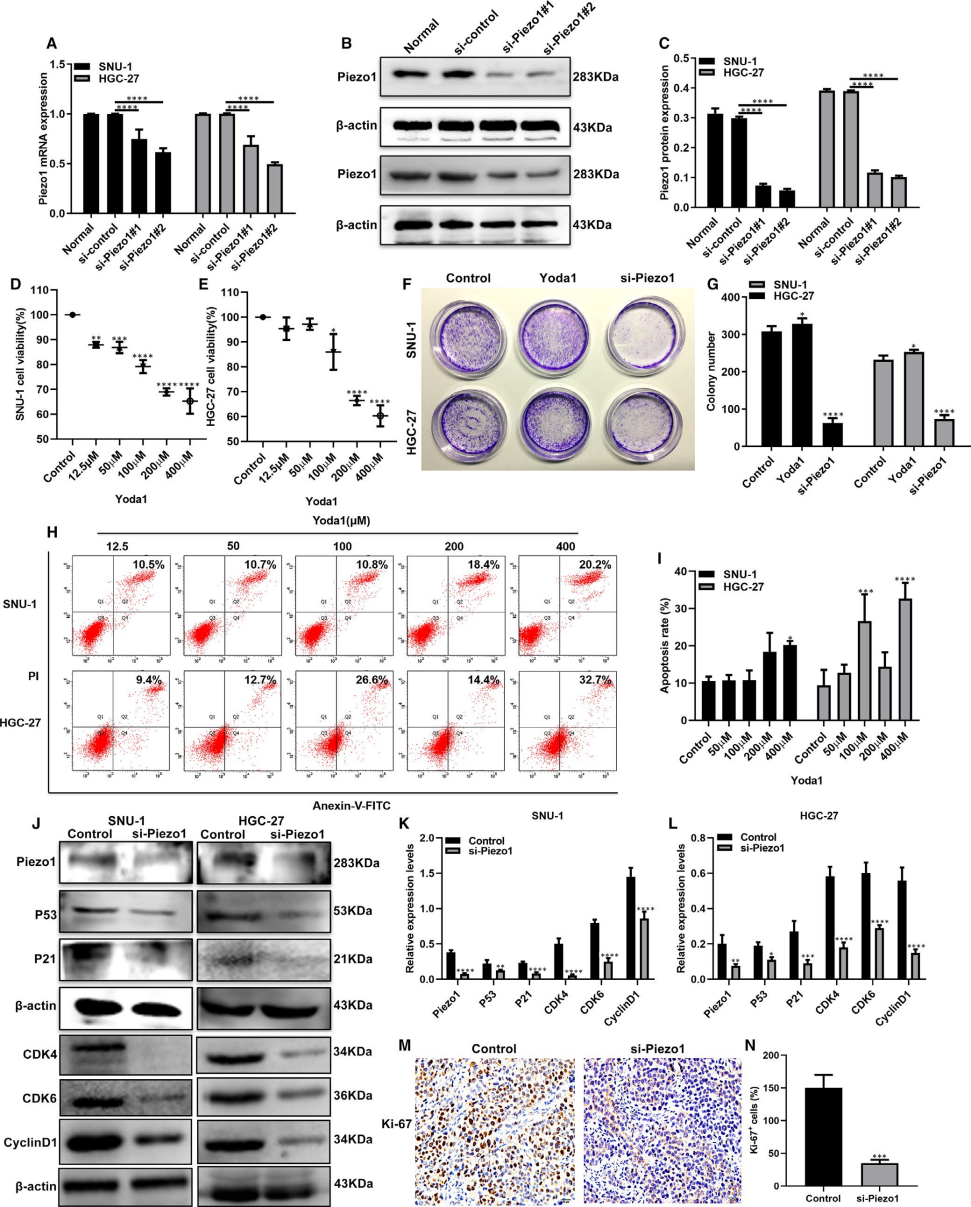
Piezo1 induces cell proliferation and inhibits cell apoptosis in GC cells. A‐C, qRT‐PCR and Western blot assays were used to detect the expression of Piezo1 in GC cells transfected with two siRNAs. D, E, CCK‐8 assay results showed the cell viability of two GC cells treated with different concentrations of Yoda1. F, G, The effects of Piezo1 on colony formation ability were observed in two GC cells transfected with si‐Piezo1 or Yoda1. H, I, Flow cytometry assay was used to detect the cell apoptosis in two GC cells treated with different concentrations of Yoda1. J‐L, Western blot was performed to detect the expression of P53, P21, CDK4, CDK6 and CyclinD1 proteins in two GC cells transfected with si‐Piezo1. M, N, Immunohistochemistry results showed the decreased Ki‐67‐positive cells following transfection with si‐Piezo1. *P < .05; **P < .01; ***P < .001; ****P < .0001

Two siRNAs targeting Piezo1 were designed and synthesized. The transfection efficiency of si‐Piezo1 was assessed in two GC cells (SNU‐1 and HGC‐27) using qRT‐PCR and Western blot. We found that Piezo1 mRNA and protein expression was prominently suppressed in two GC cells after transfection with the two siRNAs (Figure 2A‐C). Piezo1 ion channel agonist (Yoda1) was used to activate Piezo1. CCK‐8 assay results showed that the cell viability was significantly increased in two GC cells treated with Yoda1, with a dose‐dependent manner (Figure 2D, E). 50 μM Yoda1 was identified to overexpress Piezo1. As shown in the colony formation assay results, cell proliferative ability of two GC cells was significantly increased after treatment with Yoda1, which was remarkably decreased after transfection with si‐Piezo1 (Figure 2F, G). Flow cytometry assay results showed that cell apoptotic activity of GC cells was significantly decreased following treatment with Yoda1 with a concentration‐dependent manner (Figure 2H, I). The p53/p21 complex may regulate invasion and apoptosis for tumour cells.^16^ Thus, we detected the expression of p53 and p21 proteins in two GC cells transfected with si‐Piezo1 by Western blot. As shown in Figure 2J‐L, the expression of P53 and P21 proteins was distinctly decreased in GC cells after transfection with si‐Piezo1. Other cell cycle regulators including CDK4, CDK6 and CyclinD1 were also detected using Western blot. Our data suggested that CDK4, CDK6 and CyclinD1 expression was all significantly lowered following transfection with si‐Piezo1 (Figure 2J‐L). Furthermore, our immunohistochemistry results confirmed that Ki‐67‐positive cells were remarkedly decreased after treatment with si‐Piezo1 (Figure 2M, N). These results suggested that Piezo1 could facilitate cell proliferation and suppress cell apoptosis in GC cells.

### 3.3 Piezo1 promotes Ca^2+^ level and mitochondrial membrane potential in GC cells

**FIGURE 3:**
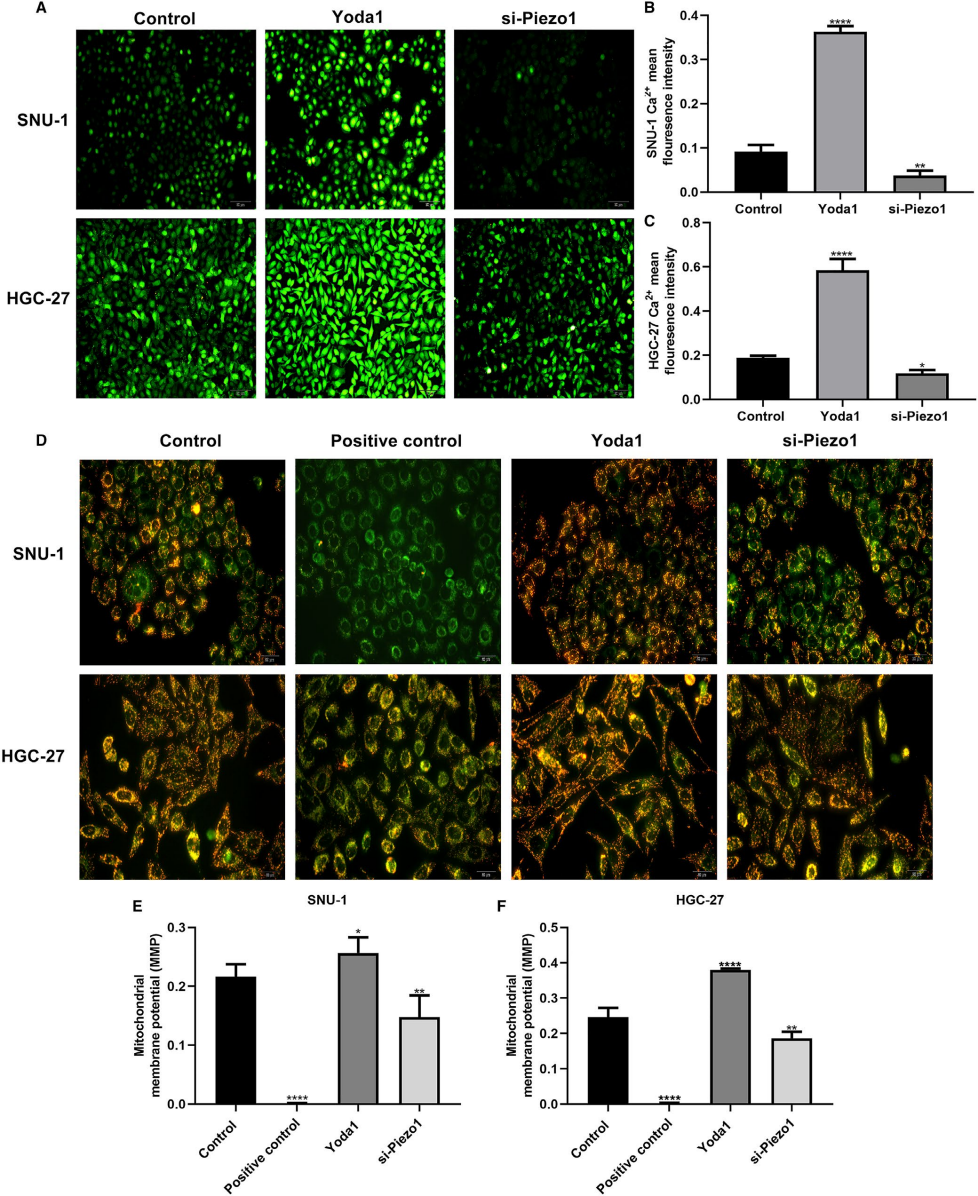
Piezo1 promotes Ca^2+^ level and mitochondrial membrane potential in GC cells. A‐C, Fluo4 assay results showed the Ca^2+^ level in SNU‐1 and HGC‐27 cells transfected with Yoda1 or si‐Piezo1. D‐F, JC‐1 assay results suggested the mitochondrial membrane potential in two GC cells after treatment with Yoda1 or si‐Piezo1. *P < .05; **P < .01; ****P < .0001

Fluo4 assay results showed that Ca^2+^ level was distinctly increased in SNU‐1 and HGC‐27 cells treated with Yoda1 (Figure 3A‐C). However, when transfected with si‐Piezo1, Ca^2+^ level was significantly decreased. As shown in JC‐1 assay, results showed that Yoda1 significantly increased the MMP (Ψm) in two GC cells, whereas si‐Piezo1 remarkably reduced the Ψm in GC cells (Figure 3D‐F). Above results suggested that Piezo1 could induce Ca^2+^ level and MMP in GC cells.

### 3.4 Piezo1 induces the expression of HIF‐1α, VEGF and EMT‐related proteins in GC cells

In SNU‐1 and HGC‐27 two GC cells, Piezo1 mRNA expression was activated by Yoda1 and inhibited by si‐Piezo1. The mRNA expression levels of HIF‐1α, VEGF and EMT‐related Vimentin and N‐cadherin were remarkedly elevated in GC cells treated with Yoda1, which were suppressed after transfection with si‐Piezo1. Furthermore, Yoda1 prominently suppressed the mRNA expression of EMT‐related E‐cadherin. Conversely, si‐Piezo1 transfection significantly elevated the mRNA expression of E‐cadherin. We also examined the expression of above proteins by Western blot. As expected, Yoda1 conspicuously boosted the expression of Piezo1, HIF‐1α, VEGF and EMT‐related Vimentin and N‐cadherin. Nevertheless, the expression of these proteins was cut down following transfection with si‐Piezo1. Consistent with qRT‐PCR results, E‐cadherin protein expression was distinctly decreased after treatment with Yoda1 in GC cells, which was elevated by si‐Piezo1 (Supplementary Figure S1).

### 3.5 Piezo1 could promote cell migration via up‐regulation of HIF‐1α in GC cells

**FIGURE 4:**
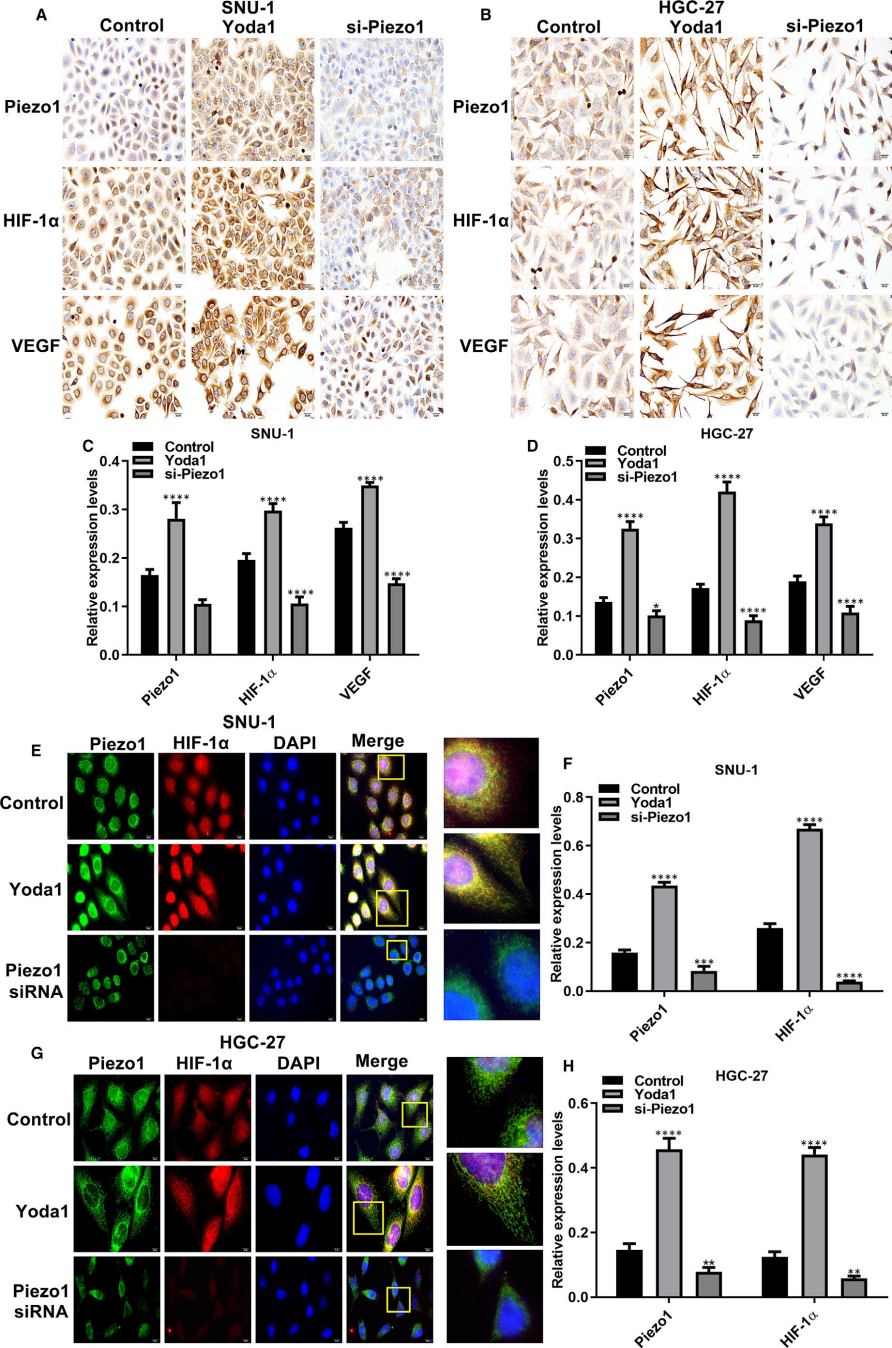
Piezo1 promotes the expression of HIF‐1α and VEGF proteins in GC cells. A, B, Representative images of immunohistochemistry for SNU‐1 and HGC‐27 two GC cells treated with Yoda1 and si‐Piezo1. C, D, The expression of Piezo1, HIF‐1α and VEGF was quantified in GC cells according to immunohistochemistry. E‐H, Immunofluorescence assay results showed the expression of Piezo1, HIF‐1α and VEGF in GC transfected with Yoda1 and si‐Piezo1. *P < .05; **P < .01; ***P < .001; ****P < .0001

**FIGURE 5:**
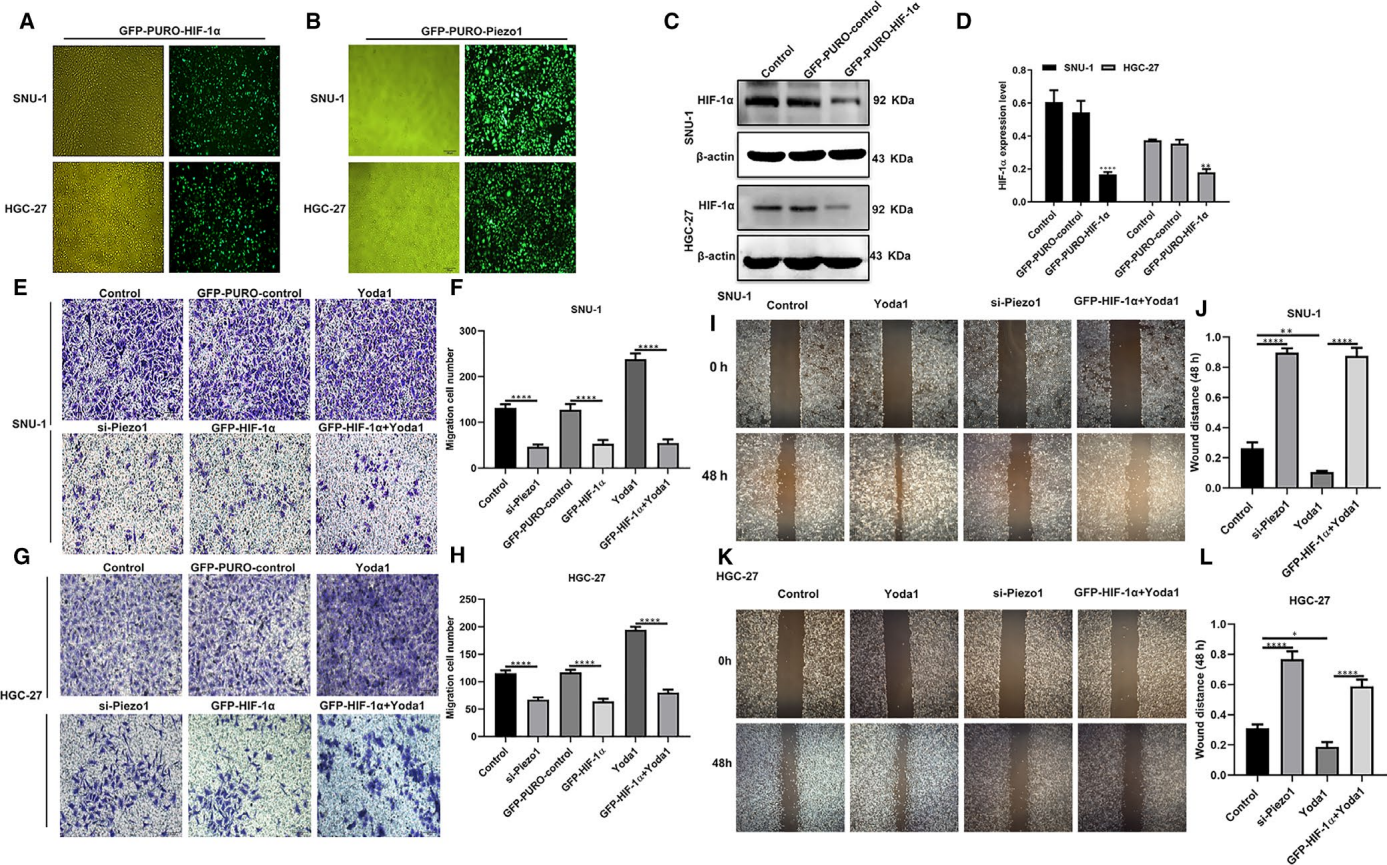
Piezo1 could promote cell migration via up‐regulation of HIF‐1α in GC cell. A, B, Representative images of SNU‐1 and HGC‐27 two GC cells stably transfected with HIF‐1α and Piezo1 knockdown. C, D, Western blot was used to assess the transfection efficiency of HIF‐1α knockdown in GC cells. E‐H, Transwell assay was performed to detect the migrant ability of GC cells after treatment with si‐Piezo1, Yoda1 or si‐Piezo1 + Yoda1. I‐L, Wound healing assay was used to measure the wound distance for GC cells transfected with si‐Piezo1, Yoda1 or si‐Piezo1 + Yoda1. *P < .05; **P < .01; ****P < .0001

We focused on the effect of dysregulated Piezo1 on HIF‐1α and VEGF expression. As expected, immunohistochemistry results suggested that Piezo1 overexpression remarkedly activated the expression of HIF‐1α and VEGF proteins in SNU‐1 and HGC‐27 two GC cells (Figure 4A‐D). In converse, their expression was suppressed following transfection with si‐Piezo1. Above results were verified by immunofluorescence (Figure 4E‐H). To explore the relationship between Piezo1 and HIF‐1α, we constructed two GC cells stably transfected with HIF‐1α and Piezo1 knockdown (Figure 5A, B). Western blot confirmed that HIF‐1α was efficiently silenced in GC cells (Figure 5C, D). Transwell results showed that the migrant ability of GC cells was distinctly suppressed by si‐Piezo1 and promoted by Yoda1 (Figure 5E‐H). Furthermore, we found that HIF‐1α also inhibited GC cell migration. Intriguingly, when co‐transfection of Yoda1 and HIF‐1α knockdown, GC cell migration was remarkedly decreased in comparison with transfection with Yoda1, indicating that silenced HIF‐1α could reverse the promotion effect of Piezo1 overexpression on GC cell migration. Wound healing assay was also carried out. Similarly, Piezo1 knockdown prominently suppressed the GC cell migration (Figure 5I‐L). In converse, Yoda1 significantly accelerated the migrant ability of GC cells. Nevertheless, after silencing HIF‐1α, the promotion effects of Piezo1 overexpression on migrant ability of GC cells were prominently weakened (Figure 5I‐L). These findings indicated that Piezo1 could promote cell migration via regulation of HIF‐1α in GC cells.

### 3.6 Piezo1 physically interacts with HIF‐1α in GC cells

We further evaluated whether Piezo1 could physically interact with HIF‐1α. Our co‐immunoprecipitation results showed that Piezo1 and HIF‐1α existed in the same protein complex in SNU‐1 and HGC‐27 cells (Supplementary Figure S2). Piezo1 knockdown decreased, whereas Piezo1 overexpression increased the interaction between Piezo1 and HIF‐1α. Hence, our data indicated that Piezo1 could physically interact with HIF‐1α.

### 3.7 Piezo1 could promote Calpain1/2 expression by up‐regulation of HIF‐1α in GC cells

Calpain1/2 are members of the Ca^2+^‐dependent neutral cysteine proteases. Our immunohistochemistry results showed that Calpain1 had distinctly higher expression levels in GC tissues in comparison with normal tissues or GC tissues with omentum metastasis. Furthermore, Calpain2 not Calpain1 was significantly up‐regulated in GC tissues with omentum metastasis compared to normal tissues or GC tissues. E‐cadherin expression was remarkedly decreased both in GC tissues and GC tissues with omentum metastasis. As shown in Supplementary Figure S3, Piezo1 knockdown prominently decreased the expression of Calpain1 and Calpain2 in GC cells according to immunohistochemistry results. Then, we performed Western blot analysis. Piezo1 overexpression was induced by Yoda1 in GC cells, which was ameliorated after co‐transfection with Yoda1 and HIF‐1α knockdown, indicating that HIF‐1α might induce the expression Piezo1 in GC cells. Also, si‐Piezo1 distinctly decreased HIF‐1α expression, whereas Piezo1 overexpression elevated its expression in GC cells. Nevertheless, when co‐transfection with HIF‐1α knockdown and Yoda1, HIF‐1α expression was significantly inhibited. Consistent with immunohistochemistry, the expression of Calpain1 and Calpain2 was remarkedly suppressed by si‐Piezo1 in GC cells, which was elevated induced by Yoda1. However, after silencing HIF‐1α, the expression of Calpain1 and Calpain2 was decreased in GC cell transfected with Yoda1 (Supplementary figure S3). Above results indicated that Piezo1 could induce Calpain1/2 expression via up‐regulation of HIF‐1α in GC cells.

### 3.8 Piezo1 knockdown could significantly inhibit peritoneal metastasis of GC

**FIGURE 6:**
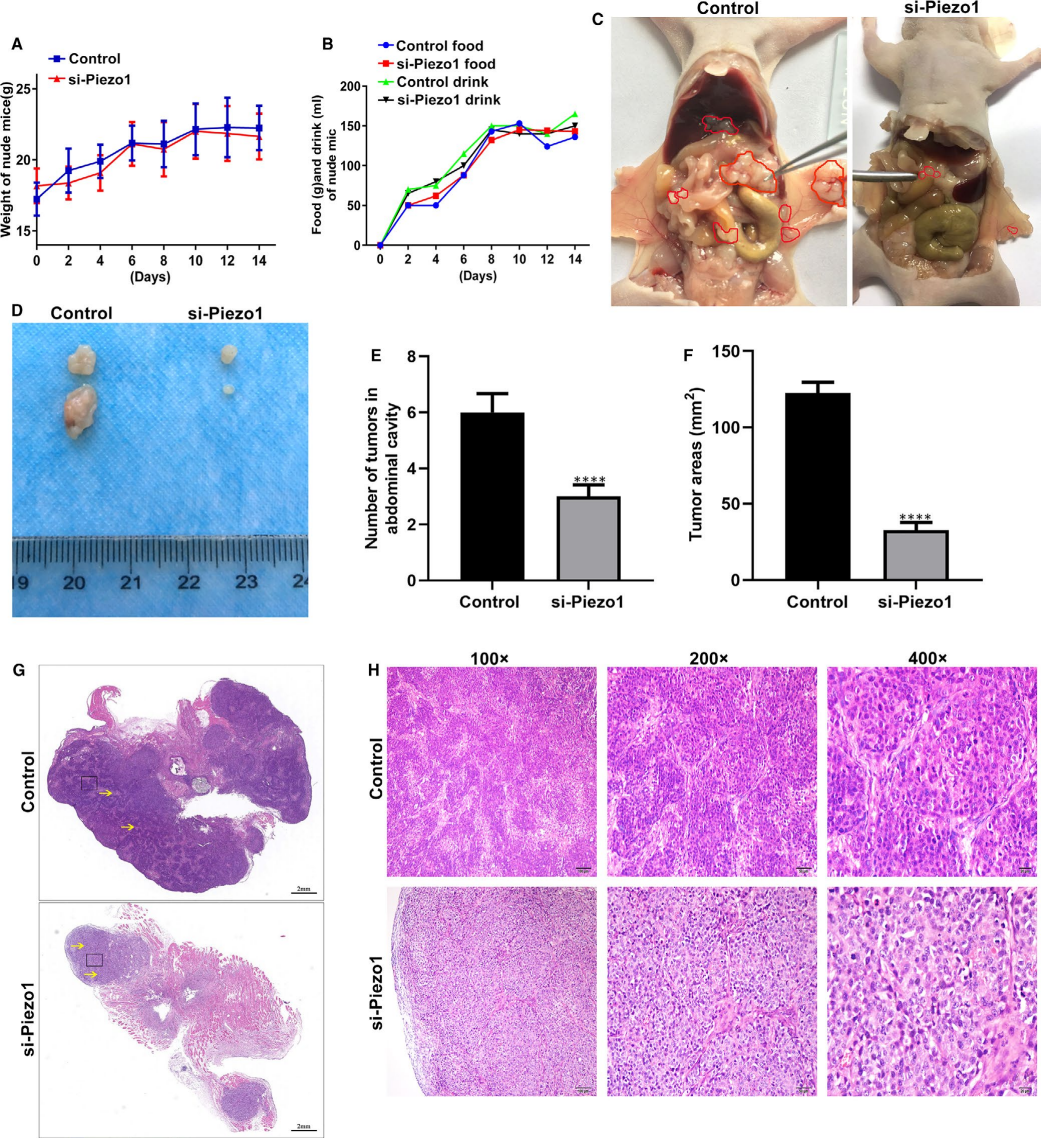
Piezo1 knockdown significantly inhibited peritoneal implantation and metastasis of GC cells. A, B, No significant differences in bodyweight and diet between the si‐Piezo1 nude mouse peritoneal metastasis of GC model group and the control model group during 14 days. C‐E, si‐Piezo1 group had lower number and volume of intraperitoneal implanted GC tumours. Red areas represent tumour tissues. F, Lower tumour areas were detected in si‐Piezo1 group in comparison with control group. G, H, H&E staining was performed to observe tumour tissue structure and tumour cell morphology changes between the two groups. Yellow arrow indicates a small clustered area). Magnification: 100×; 200×; 400×. ****P < .0001

The peritoneal metastasis of GC model was established in nude mice. No significant differences in bodyweight or diet were observed after nude mouse models injected with si‐Piezo1 or control during 14 days (Figure 6A, B). si‐Piezo1 remarkedly reduced the number and volume of intraperitoneal implanted GC tumours in nude mice (Figure 6C‐E). Haematoxylin & eosin (H&E) staining results showed that the tumour area in the si‐Piezo1 group was significantly decreased than that in the control group (Figure 6F). Furthermore, the peritoneal tumour tissues in the control group had tight structures with dark nuclear staining and small clusters of tumour cell aggregation. In the si‐Piezo1 group, the tumour cells had a loose structure, light nuclear staining, nuclear pyknosis in some cells and were scattered (Figure 6G, H). Our data results demonstrated that Piezo1 knockdown significantly inhibited peritoneal implantation and metastasis of GC cells.

### 3.9 Piezo1 knockdown could block EMT process and angiogenesis in peritoneal metastatic GC

**FIGURE 7:**
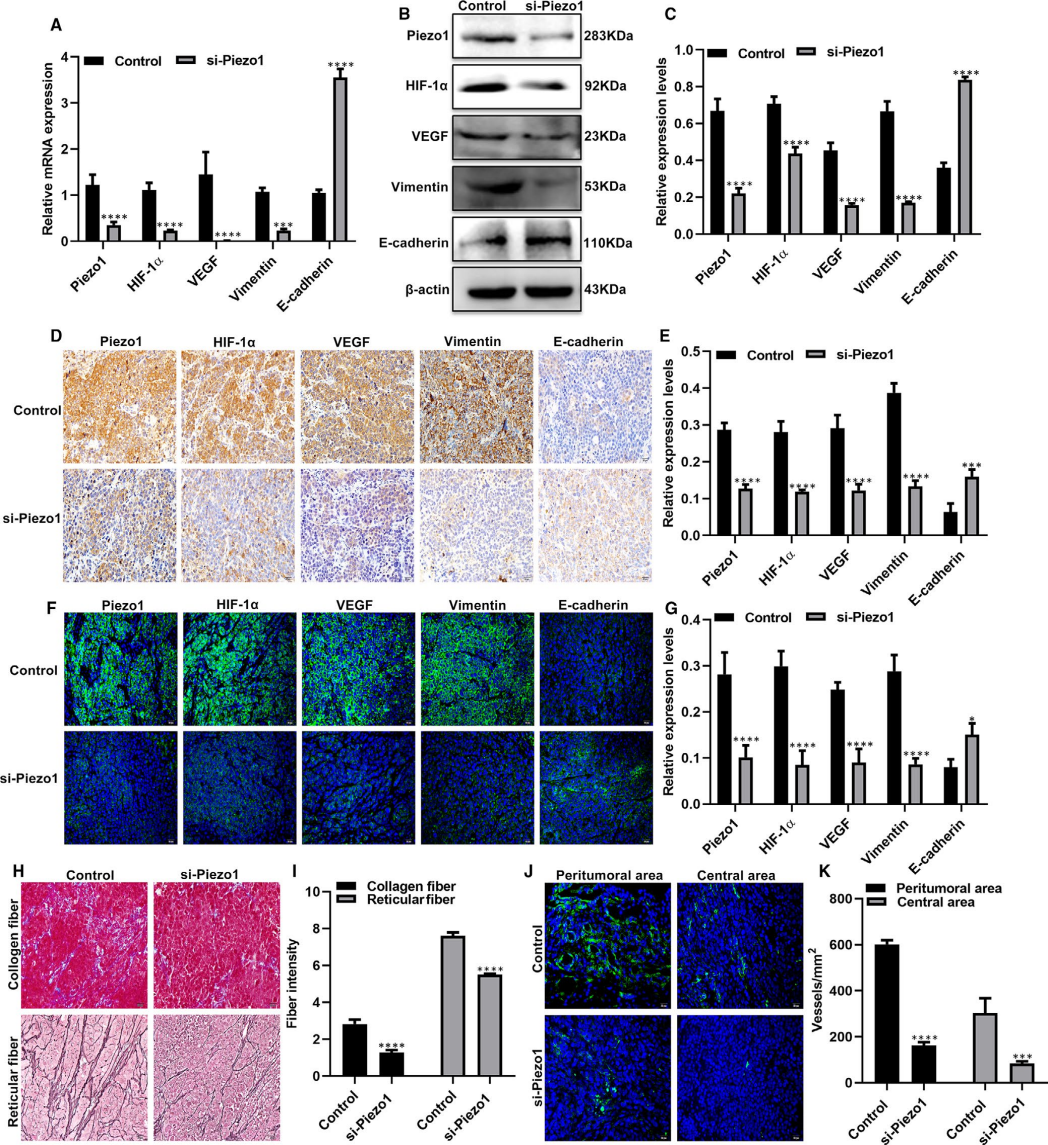
Piezo1 knockdown could block EMT process and angiogenesis in peritoneal metastatic GC. A‐C, qRT‐PCR and Western blot assays were used to detect the mRNA and protein expression levels of Piezo1, HIF‐1α, VEGF, vimentin and E‐cadherin in peritoneal metastatic tumour tissues of nude mouse models after transfection with si‐Piezo1. D‐G, The expression of Piezo1, HIF‐1α, VEGF, vimentin and E‐cadherin proteins was quantified by immunohistochemistry and immunofluorescence. H, I, Masson's trichrome staining and reticular fibre staining were performed to observe intratumoural collagen fibres (blue) and reticular fibres (black). J, K, The vascular density in the peritoneal tumour tissues of was determined by immunofluorescence. *P < .05; ***P < .001; ****P < .0001

qRT‐PCR and Western blot assays were used to examine the expression levels of Piezo1, HIF‐1α, VEGF, vimentin and E‐cadherin in peritoneal metastatic GC tumour tissues. As shown in Figure 7A‐C, after transfection with si‐Piezo1, the expression levels of Piezo1, HIF‐1α, VEGF and vimentin were notably decreased, whereas the mRNA expression levels of E‐cadherin were significantly elevated in peritoneal metastatic tumour tissues at the mRNA and protein levels. Similar results were observed in immunohistochemistry and immunofluorescence (Figure 7D‐G). si‐Piezo1 significantly reduced collagen fibre and reticular fibre formation in peritoneal metastatic tumour tissues (Figure 7H, I). We also found that both in the central areas and peritumoural areas of peritoneal tumour tissues, CD34‐labelled vascular density was prominently reduced in si‐Piezo1 group than that in control group (Figure 7J, K). These results indicated that Piezo1 knockdown not only blocked EMT processes but also angiogenesis in peritoneal metastatic tumour tissues. Potential mechanisms of Piezo1 on GC omentum metastasis were shown in Supplementary figure S4.

## 4 DISCUSSION

In this study, highly expressed Piezo1 was found both in GC tissues with or without omentum metastasis. Studies have shown that Piezo1 promotes the proliferation and migration of GC cells.^15^ Thus, we studied the role and mechanism of Piezo1 in the omentum implantation and metastasis of GC cells in depth.

We analysed the relationship between Piezo1 expression and clinical characteristics of GC. The results suggested that Piezo1 was closely related to lymph metastasis, TNM stage and distant metastasis. High Piezo1 expression usually predicted a poor prognosis. In addition, omentum metastatic lesions showed higher Piezo1 expression. Piezo1 could promote cell proliferation and inhibit apoptosis in GC cells. Ψm could regulate various basic cell processes, including cell proliferation, gene transcription, differentiation and cell death.^17^ Furthermore, Ψm plays a key role in cancer metastasis and angiogenesis.^18^ In this study, Piezo1 activation increased the Ψm and Ca^2+^ levels in GC cells.^19^, ^20^, ^21^ However, after Piezo1 interference, the Ψm and Ca^2+^ levels in GC cells were significantly decreased. Furthermore, we examined the effect of Piezo1 interference on the P53/P21 axis, a key signal of proliferation.^22^, ^23^ We found that the expression of the P53/P21 axis was also significantly decreased. Notably, Piezo1 might be a key factor that regulates GC cell proliferation and might function by regulating Ψm.

Herein, GC cells were transfected with si‐Piezo1 to stimulate HIF‐1α expression and we observed whether HIF‐1α expression could affect Piezo1 expression. We found that under Piezo1 knockdown conditions, HIF‐1α mRNA and protein expression levels were prominently reduced. Similarly, HIF‐1α silencing attenuated the expression levels of Piezo1 mRNA and protein in GC cells. It has been confirmed that Piezo1 induced the expression of HIF‐1α in colon cancer cells.^24^ Our co‐immunoprecipitation confirmed the interactions between HIF‐1a and Piezo1 in GC cells. Silenced Piezo1 significantly suppressed the migration ability of GC cells. However, the migration of GC cells was notably promoted by Piezo1 activation induced by Yoda1, which was reversed by HIF‐1α knockdown. Therefore, Piezo1 may induce the cells to adapt to the hypoxic environment and accelerate the migration and metastasis of tumour cells by up‐regulation of HIF‐1α.^25^, ^26^, ^27^ This discovery requires more in‐depth experimental and clinical verification.

Numerous studies have shown that EMT is an important early process of invasion and metastasis for GC cells.^28^, ^29^, ^30^ The ability of tumour cells to spread to the surrounding and invade normal tissues is the key to the evolution of tumour cells into malignant tumours. By detection of EMT‐related proteins, our findings suggested that Piezo1 accelerated the EMT process in GC cells. To further verify the effect of Piezo1 on the abdominal cavity implantation of GC, nude mice were intraperitoneally injected with GC cells. Our animal experiments showed that inhibition of Piezo1 can prevent the tumour growth in omentum and peritoneal implantation. For the control group, although the HGC‐27 cells were injected, the number of tumour nodules increased significantly with time. Tumour nodules firstly appear in the omentum. Compared with the control group, the number and volume of tumour nodules on the omentum of nude mice in the si‐Piezo1 group were significantly reduced. The mRNA and protein expression levels of Piezo1, HIF‐1α, VEGF and Vimentin in the peritoneal tumour tissues in the si‐Piezo1 group were significantly lower than those in the control group. The expression of E‐cadherin in the si‐Piezo1 group was remarkedly higher than that in the control group. Furthermore, the collagen fibre and reticular fibre of the experimental group were significantly reduced. More importantly, si‐Piezo1 significantly inhibited angiogenesis in peritoneal metastatic GC. Studies have shown that HIF‐1α expression is related to tumour invasion depth. In invasive tumours, microvessel density is significantly correlated with CD163‐positive macrophages and lymph node metastasis in the corresponding region.^31^, ^32^ Our findings indicated that Piezo1 might promote GC peritoneal metastasis by up‐regulation of HIF‐1α. Thus, Piezo1 overexpression may aggravate the hypoxic state of local tumours and facilitate tumour growth, ultimately accelerating peritoneal implantation and metastasis of GC.

In summary, the above results indicated that Piezo1 overexpression can up‐regulate HIF‐1α expression. Piezo1 knockdown can reduce the expression of Piezo1 and HIF‐1α in GC cells and omentum metastatic tumour tissues, thereby effectively inhibiting the EMT process of GC cells as well as the peritoneal cavity implantation and metastasis of GC cells in nude mice. Thus, Piezo1 could become an underlying treatment target for GC peritoneal metastases.

## 5 CONCLUSION

Piezo1 was highly expressed in GC tissues with omentum metastasis. The up‐regulation could accelerate cell migration and Calpain1/2 expression by up‐regulation of HIF‐1α in GC cells. In peritoneal metastatic GC mouse model, we found that silencing Piezo1 could notably suppress peritoneal metastatic tumour growth, block EMT process and angiogenesis. Thus, Piezo1 could become a therapeutic target for GC patients with omentum metastasis, which requires to be validated by clinical and basic experiments.

## CONFLICT OF INTEREST

The authors declare no conflicts of interest.

## AUTHOR CONTRIBUTIONS

**Xiaofei Wang:** Conceptualization (equal); Data curation (equal); Formal analysis (equal); Funding acquisition (equal); Writing‐review & editing (equal). **Guang Cheng:** Investigation (equal); Methodology (equal); Visualization (equal); Writing‐original draft (equal). **Yu Miao:** Methodology (equal); Software (equal); Visualization (equal); Writing‐original draft (equal). **Fangyuan Qiu:** Resources (equal); Software (equal); Validation (equal). **Lugen Bai:** Formal analysis (equal); Visualization (equal); Writing‐original draft (equal). **Zhongfei Gao:** Resources (equal); Software (equal); Supervision (equal). **Yunning Huang:** Data curation (equal); Formal analysis (equal); Writing‐original draft (equal). **Liru Dong:** Investigation (equal); Validation (equal). **Xing Niu:** Conceptualization (equal); Data curation (equal); Writing‐original draft (equal); Writing‐review & editing (equal). **Xin Wang:** Investigation (equal); Software (equal); Validation (equal). **Yuyang Li:** Visualization (equal); Writing‐original draft (equal). **Hui Tang:** Software (equal); Visualization (equal). **Yuanyi Xu:** Conceptualization (equal); Data curation (equal); Formal analysis (equal); Project administration (equal); Writing‐review & editing (equal). **Xudong Song:** Conceptualization (equal); Data curation (equal); Project administration (equal); Supervision (equal); Writing‐review & editing (equal).

## ETHICS APPROVAL AND CONSENT TO PARTICIPATE

The study was approved by the Ethics Committee of North China University of Science and Technology Affiliated Hospital (2014025).

## CONSENT FOR PUBLICATION

All patients were informed consent.

## Supporting information

Fig S1

Fig S2

Fig S3

Fig S4

## Data Availability

The data sets analysed during the current study are available from the corresponding author on reasonable request.
