# Supplementary figures and images for: Piezo type mechanosensitive ion channel component 1 facilitates gastric cancer omentum metastasis
Source: J Cell Mol Med. 2021 Jan 13;25(4):2238–53. doi: 10.1111/jcmm.16217 (PMC7882944; doi:10.1111/jcmm.16217)

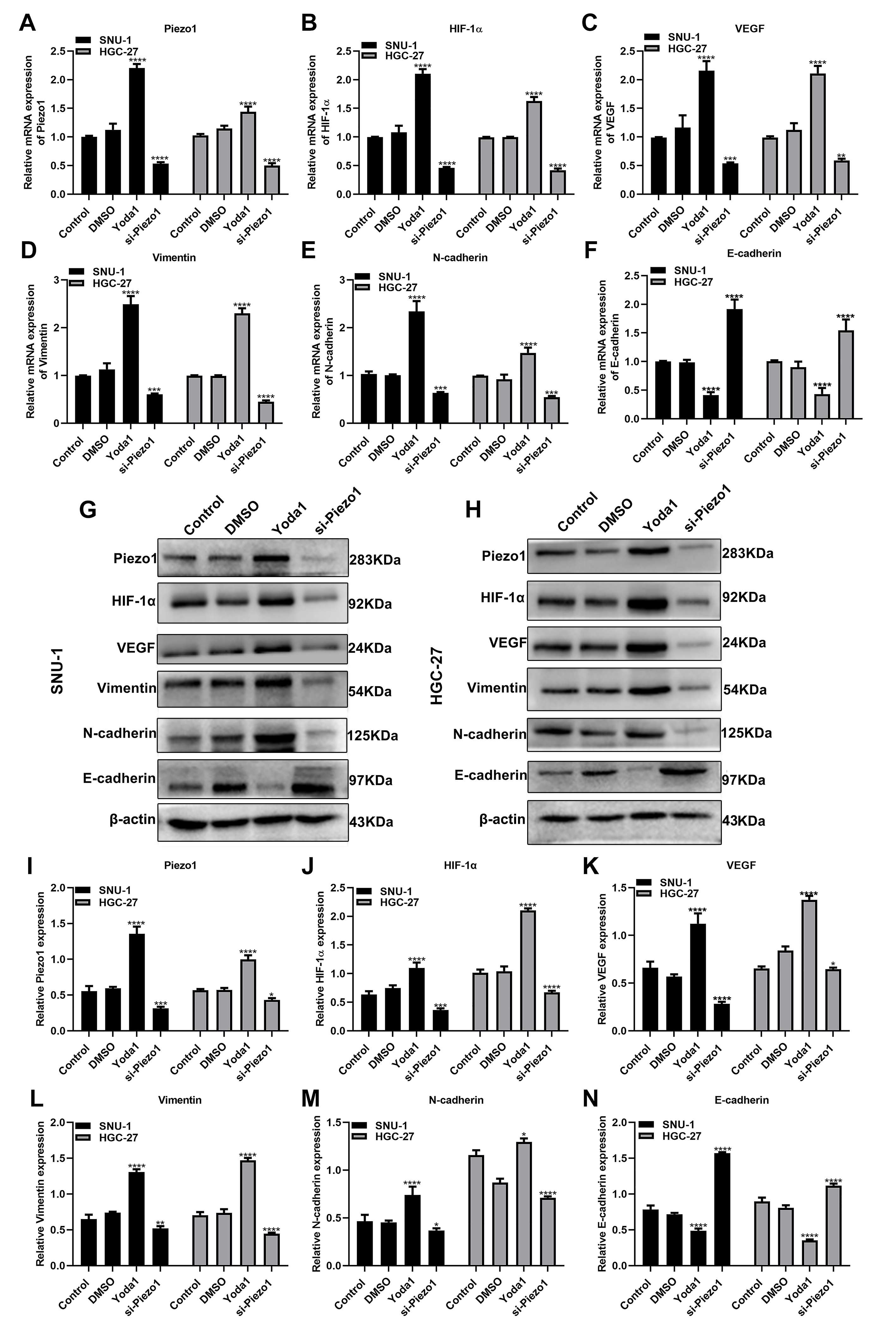

Supplement: Supplementary file 1 — Fig S1 [file JCMM-25-2238-s001.tif]

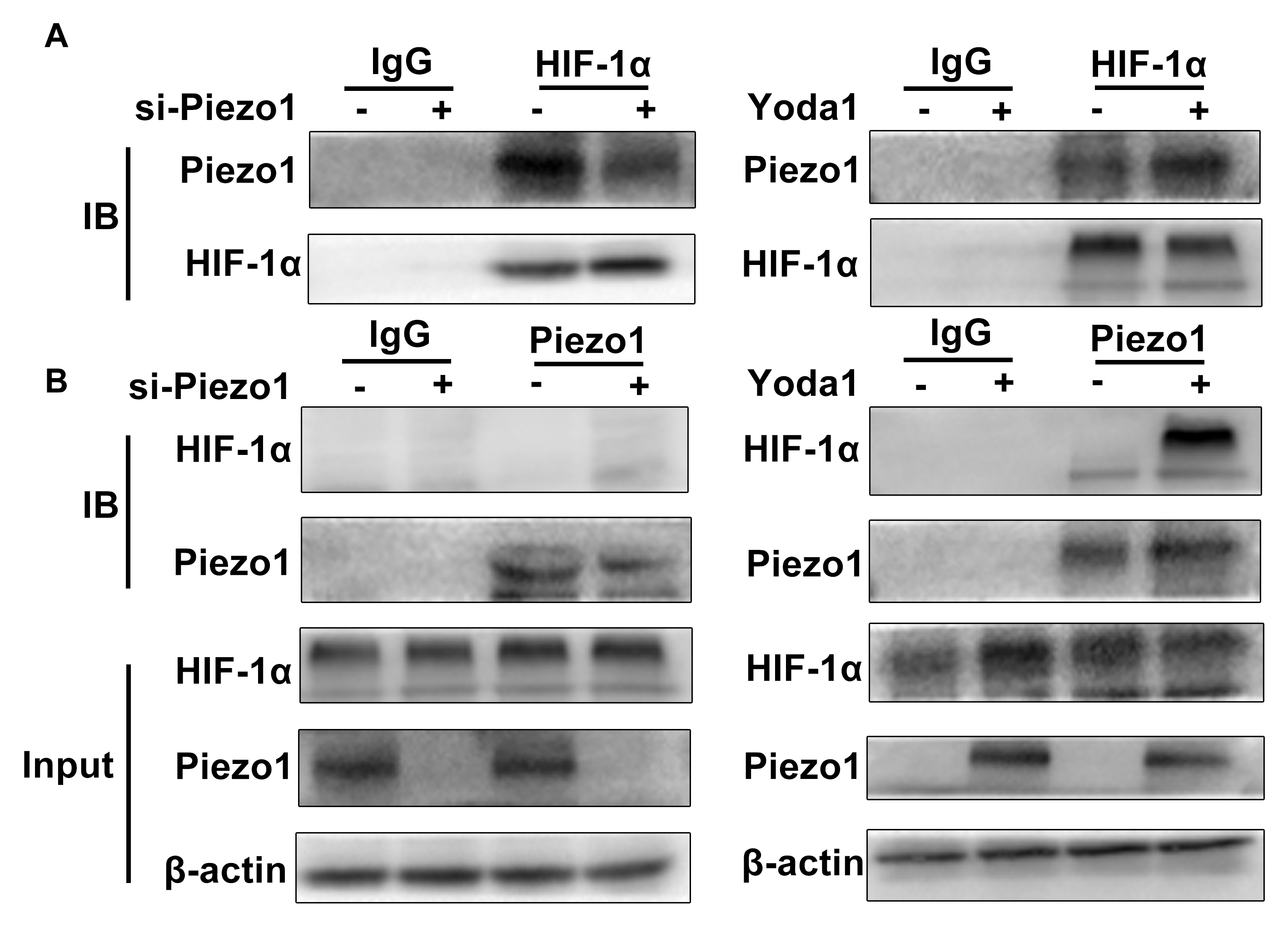

Supplement: Supplementary file 2 — Fig S2 [file JCMM-25-2238-s002.tif]

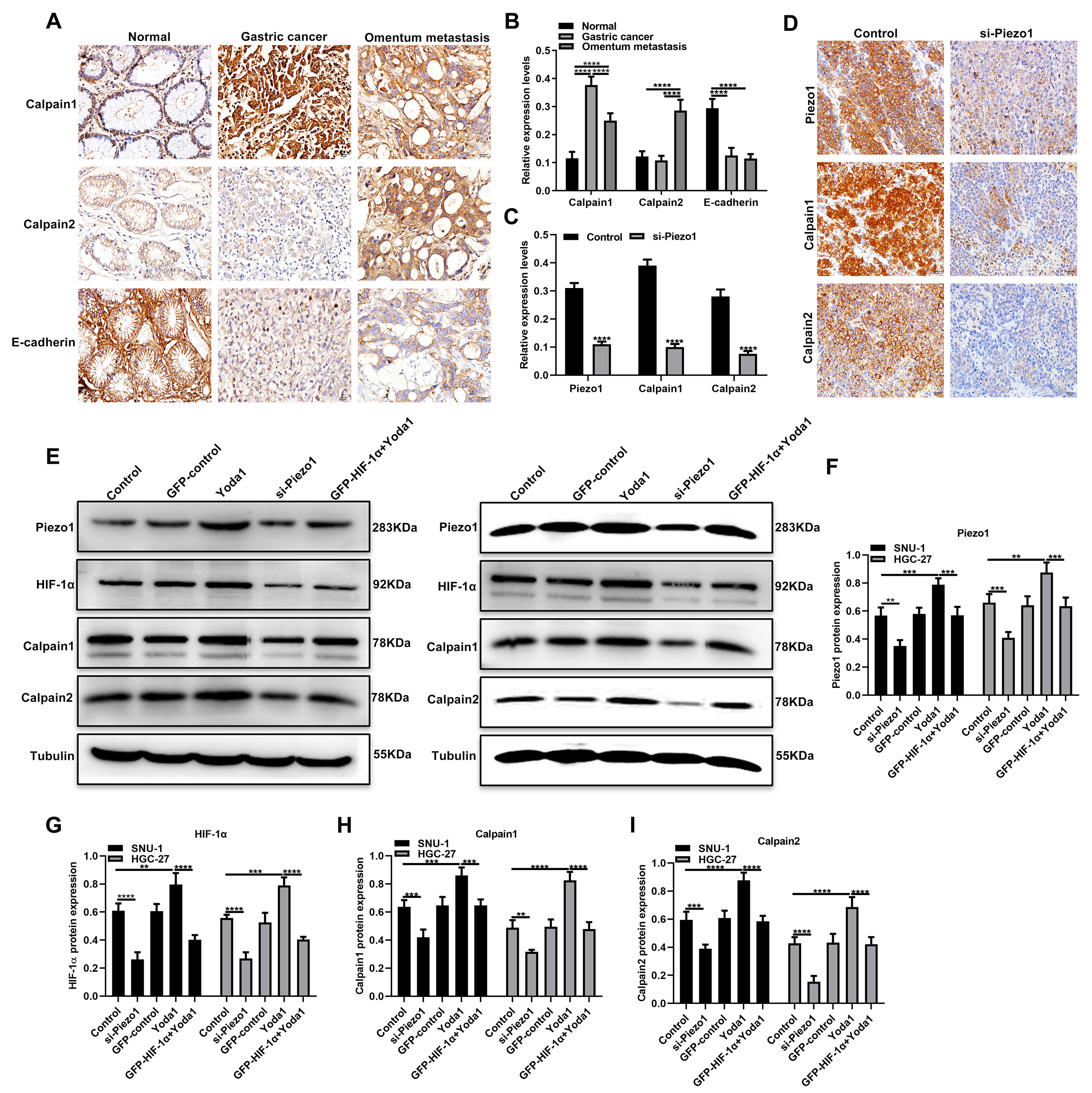

Supplement: Supplementary file 3 — Fig S3 [file JCMM-25-2238-s003.tif]

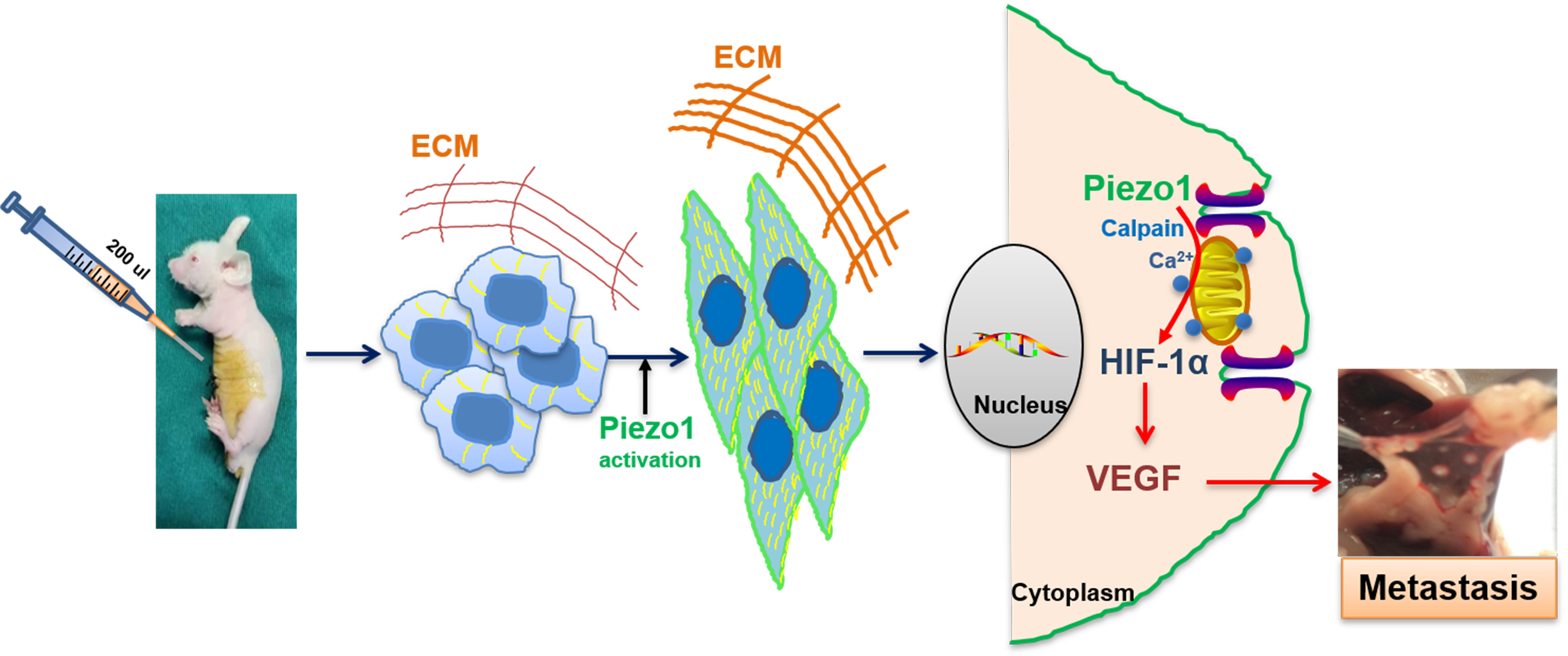

Supplement: Supplementary file 4 — Fig S4 [file JCMM-25-2238-s004.tif]
